# Decoding Tumor Angiogenesis for Therapeutic Advancements: Mechanistic Insights

**DOI:** 10.3390/biomedicines12040827

**Published:** 2024-04-09

**Authors:** Geetika Kaur, Bipradas Roy

**Affiliations:** 1 Integrative Biosciences Center, Wayne State University, Detroit, MI 48202, USA; geetikakaur18@gmail.com; 2 Department of Ophthalmology, Visual and Anatomical Sciences, Wayne State University School of Medicine, Detroit, MI 48202, USA; 3 Division of Cardiology, Department of Medicine, Duke University Medical Center, Durham, NC 27710, USA

**Keywords:** tumor angiogenesis, proangiogenic factors, antiangiogenic factors, vascular endothelial growth factor, fibroblast growth factor, platelet-derived growth factor, angiopoietin, matrix metalloproteases, interleukins, endostatin

## Abstract

Tumor angiogenesis, the formation of new blood vessels within the tumor microenvironment, is considered a hallmark of cancer progression and represents a crucial target for therapeutic intervention. The tumor microenvironment is characterized by a complex interplay between proangiogenic and antiangiogenic factors, regulating the vascularization necessary for tumor growth and metastasis. The study of angiogenesis involves a spectrum of techniques, spanning from biomarker assessment to advanced imaging modalities. This comprehensive review aims to provide insights into the molecular intricacies, regulatory dynamics, and clinical implications of tumor angiogenesis. By delving into these aspects, we gain a deeper understanding of the processes driving vascularization in tumors, paving the way for the development of novel and effective antiangiogenic therapies in the fight against cancer.

## 1. Introduction

Angiogenesis is a complex physiological process, involving the formation of new blood vessels from pre-existing vessels. It plays a pivotal role in embryonic development, survival of neoplasms, wound healing, and tissue repair [1,2,3]. It is a continuous process occurring across the lifespan, initiating in utero, and persisting into advanced age. The formed blood vessels are essential for facilitating the exchange of nutrients and metabolites through diffusion [4,5]. Oxygen plays a central role in regulating this process, while hemodynamic factors are crucial for sustaining vascular networks and shaping vessel wall structures [6]. In healthy tissues, blood vessels grow dynamically, and regress based on functional requirements and demands. During the past four decades, angiogenesis has sparked significant interest due to its therapeutic potential. For instance, angiogenesis proves beneficial in managing pathological conditions such as cancer, diabetic retinopathy, heart diseases, atherosclerosis, wound healing, and rheumatoid arthritis [7,8,9].

**Figure 1:**
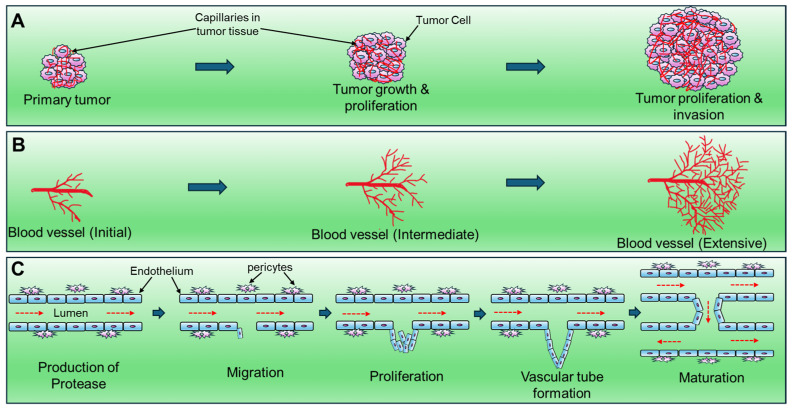
Stages of Angiogenesis in tumor growth. (**A**) Association of blood vessel formation in different stages of tumor growth. (**B**) Formation of new blood vessels from pre-existing blood vessels. (**C**) Different stages of angiogenesis. Broken red arrows denote the direction of blood flow in the lumen, and thick arrows indicate the next steps.

Angiogenesis is an intricate process that involves several distinct stages, including initiation, migration, proliferation, vascular tube formation, and maturation, as depicted in Figure 1C [10]. The sequential steps in the creation of novel blood vessels encompass the production of angiogenic factors within endothelial cells (ECs), residing on the walls of existing small blood vessels. These factors are subsequently released into the microenvironment. Subsequently, these angiogenic factors bind to specific receptors on the surface of ECs, triggering a cascade of events. Upon binding, ECs become activated and release enzymes, particularly matrix metalloproteinases (MMPs), which play a vital role in breaking down the extracellular matrix (ECM) surrounding the blood vessels [11,12]. This enzymatic action facilitates the invasion of ECs into the matrix, their division, and subsequent proliferation. The activated ECs organize into strings, forming hollow tubes that intricately create new networks of blood vessels. In cancer, pericyte cells are recruited, providing essential support for the newly formed blood vessels [13,14].

Notably, angiogenesis is the hallmark feature of tumor development as it enables the sustained expansion, uncontrolled growth, and proliferation of cancerous cells [15,16]. In the 1970s, Dr. Judah Folkman proposed several hypotheses emphasizing the pivotal role of tumor angiogenesis in the initiation and metastatic dissemination of tumors. His hypotheses suggested that primary solid tumors undergo an avascular and dormant growth phase with a maximum size of approximately 1–2 mm. These microscopic tumor masses can trigger angiogenesis, thus resulting in the expansion of hematogenous metastatic spread [17,18]. The angiogenic switch was activated by tumor cells producing a growth factor known as ‘tumor angiogenesis factor’ (TAF). Tumor angiogenesis can be disrupted by inhibiting TAF production that impacts tumor growth. This therapeutic approach is referred as ‘dormancy-inducing’ and aims to control the disease by preventing new tumor expansion or causing regressions [10,19]. He speculated on the therapeutic potential of inhibiting angiogenesis as an innovative approach to treating cancer. The ideas of Folkman were influenced by studies of Algire and Chalkley, Greenblatt and Shubik, and Warren. Shubik coined the term ‘tumor angiogenesis’ [20,21].

Tumor angiogenesis is regulated by a complex interplay of proangiogenic and antiangiogenic factors [22]. Tumor cells release signaling molecules, such as VEGF and FGF, that activate endothelial cell proliferation. This leads to the formation of an intricate network of blood vessels in the tumor vicinity [23,24]. Tumor cells exhibit the ability to form elongated, tube-like structures resembling blood vessels when influenced by external factors such as growth factors or hypoxia. This phenomenon is known as vasculogenic mimicry [25]. Further, the newly formed blood vessels in tumors also undergo structural abnormalities, including irregular branching and increased permeability [26,27]. These abnormalities contribute to the inefficient blood flow within the tumor microenvironment, fostering an environment conducive to cancer cell invasion and metastasis. In addition, the abnormal vasculature can create a hypoxic environment, triggering the further release of proangiogenic signals and promotes metastasis [28,29]. Therefore, it is imperative to understand the underlying mechanisms and factors affecting tumor angiogenesis crucial for developing targeted therapies. This review provides valuable insights into the role of tumor angiogenesis in therapeutic advancements and may pave a way for innovative approaches in cancer treatment.

## 2. Possible Molecular Mechanisms of Tumor Angiogenesis: Angiogenic Switch

Angiogenesis is a multifaceted process that involves a complex interplay of cellular and molecular events. The intricate mechanism governing tumor angiogenesis involves a phenomenon called the angiogenic switch [30,31]. Under normal physiological conditions, angiogenesis is tightly regulated by a dynamic balance between proangiogenic and antiangiogenic factors which get disrupted in the tumor microenvironment. The process of angiogenesis initiates from basement membrane degradation by proteases to ECs migration into the interstitial space, the proliferation of ECs at the migrating tip, and lumen formation with the recruitment of pericytes, thus leading to blood flow [32,33]. In microvasculature, the angiogenic response involves changes in cellular adhesive interactions between adjacent ECs, pericytes, and the surrounding ECM. Active neovascularization includes the reorganization of ECs’ cytoskeleton, expression of cell surface adhesion molecules like integrins and selectins, secretion of proteolytic enzymes, and remodeling of the adjacent ECM, culminating in the formation of capillary buds [34].

Autocrine and/or paracrine angiogenic factors play a crucial role in inducing EC proliferation, migration, orientation, elongation, and differentiation, leading to the re-establishment of the basement membrane, lumen formation, and anastomosis with other vessels [35]. The angiogenic phenotype significantly contributes to the development of malignant tumors at multiple stages, where tumor cells may overexpress positive regulators of angiogenesis, mobilize angiogenic proteins from the ECM, recruit host cells like macrophages (producing their own angiogenic proteins), or engage in a combination of these processes [36].

Tumor angiogenesis is mediated by secreted angiogenic growth factors, such as VEGF and bFGF, interacting with surface receptors on ECs. This interaction initiates several signaling proteins, including PI3-kinase, Src, Grb2/m-SOS-1, and signal transducers and activators of transcriptions (STATs), each containing src-homology-2 (SH-2) domains [37]. The activation of pathways crucial for triggering the cell cycle machinery, such as the Ras-MAPK cascade, follows the binding of these proteins to receptor tyrosine kinases (RTKs). The balance between positive and negative regulators in the local milieu determines whether neovessels persist as capillaries, differentiate into mature venules or arterioles, or regress due to a preponderance of angiostatic factors, inducing apoptosis or cell cycle arrest of ECs [38].

Furthermore, small dormant tumors devoid of active blood vessel formation are often observed in early stages of cancer progression. Tumor vascularization can occur through the co-option of pre-existing vasculature or induction of new blood vessels through various molecular and cellular mechanisms [39]. The angiogenic switch, marked by a dominance of proangiogenic signaling, releases tumors from dormancy, initiating rapid growth in association with new blood vessel formation. Genetically engineered mouse models, like the RIP1-Tag2 model of pancreatic insulinoma, have been instrumental in exploring the angiogenic switch, providing insights into the diverse factors and cellular mechanisms that initiate vessel formation in tumors [40]. The switch can be triggered by additional genetic alterations, increased proliferation, hypoxia, expression of proangiogenic factors, or tumor-associated inflammation and immune cell recruitment [41]. Figure 1 depicts the possible cell signaling pathways that dictate tumor angiogenesis.

### 2.1. Proangiogenic Factors in Tumor Microvasculature

#### 2.1.1. Vascular Endothelial Growth Factor (VEGF)

VEGF, also known as vascular permeability factor (VPF), is a heparin-binding protein that plays a crucial role in tumor biology. It is involved in various crucial processes such as inducing angiogenesis, stimulating the growth and proliferation of ECs, preventing apoptosis of ECs, regulating vascular permeability, and promoting angiogenesis in tumors, chronic inflammation, and wound healing [42]. Structurally, the human VEGF gene comprises eight exons and seven introns and exists in four different isoforms: VEGF121, VEGF165, VEGF189, and VEGF 206 [43]. VEGF 145 and VEGF 183 are less frequent spliced isoforms. The most prevalent isoform is VEGF 165, which is a homodimer with a molecular mass of 45 kD [44].

The VEGF family comprises closely related factors such as VEGF-A (VEGF), VEGF-B, VEGF-C, VEGF-D, VEGF-E, and placental growth factor (PlGF). These factors signal through three tyrosine kinase receptors: VEGFR1, VEGFR2, and VEGFR3 [45]. VEGF receptors function as RTKs with extracellular regions formed by seven immunoglobulin-like domains and intracellular regions exhibiting tyrosine kinase activity [46]. In cancer, tumor cells and the stroma release VEGF, thereby playing a role in advancing tumor development, enhancing vessel density, promoting invasiveness, facilitating metastasis, and tumor recurrence [47].

Hypoxia emerges as a critical factor that triggers the expression of VEGF. Additionally, other agents such as EGF, TGF-α & β, IGF-1, FGF, and PDGF are known inducers. The hypoxia-induced transcription of VEGF mRNA is mediated by hypoxia-inducible factor-1 (HIF-1), whose binding site is located in the VEGF promoter region [48]. During hypoxia, upregulated levels of VEGF regulate blood vessel formation primarily through the activation of VEGF receptor-2 (VEGFR2), expressed by ECs, thereby leading to proliferation, cell survival, invasion, migration, and tube formation [49].

Additionally, VEGF regulates vascular permeability by various mechanisms such as fenestrae induction, junctional remodeling, and vesiculo-vascular organelles (VVOs). Evidence in the literature revealed that VEGF induces vascular permeability by mitogen-activated protein (MAP) kinase signal transduction cascade, PKB/Akt activation, and endothelial nitric-oxide synthase (eNOS) [50,51]. Furthermore, VEGF is associated with vascular inflammation, as it has the capacity to trigger the activation of the transcription factor NFAT in ECs, regulating an inflammatory gene expression pattern via PLCγ/calcineurin. VEGF regulates a proteolytic system that remodels essential extracellular matrix (ECM) components [52]. It stimulates ECs to produce urokinase-like plasminogen activator (uPA), tissue-type plasminogen activator (tPA), and plasminogen activator inhibitor-1 (PAI-1), as well as other proteolytic enzymes and factors. This activation leads to ECM breakdown, facilitating angiogenesis [53]. Additionally, VEGF induces a survival mechanism in ECs and promotes their migration and proliferation through MAPK/ERK and PI3K/Akt pathways [3,54]. PlGF, a member of the VEGF family, has a controversial role in tumor angiogenesis, with reports suggesting both proangiogenic and antiangiogenic properties. The efficacy of anti-PlGF therapy in inhibiting angiogenesis and halting tumor growth remains a subject of contradictory results in preclinical tumor models [55].

#### 2.1.2. Fibroblast Growth Factor (FGF)

The members of the FGF family comprise 23 different proteins classified into six distinct groups. Among them, 18 molecules interact with tyrosine kinase receptors viz. FGFR1, FGFR2, FGFR3, and FGFR4 [56]. All the members, notably FGF2, play a pivotal role in tumor angiogenesis by upregulating the proliferation and migration of ECs. The FGF signaling pathway intricately intersects with the VEGF signaling pathway, contributing to a complex network of interactions. The role of bFGF has been implicated in the expression of antiapoptotic proteins viz. bcl-XL and bcl-2 through the MEK/ERK signaling pathway. Overexpressed bFGF upregulates mRNA levels of VEGF in tumors, vascular smooth muscle cells, and receptors in microvascular ECs [57,58]. However, treatment with a neutralizing monoclonal antibody (mAb) against KDR/Flk-1 significantly inhibits bFGF-induced tumor development. The findings from these studies suggest that bFGF collaboratively enhances VEGF-mediated hepatocellular carcinoma development and angiogenesis by upregulating VEGF via KDR/Flk-1 [59].

Similar to VEGF, FGFs also play a crucial role in ECM degradation and organization by upregulating the production of MMPs [60]. FGF2 exerts its proangiogenic effects on ECs through paracrine signaling. FGF2, in association with VEGF, promotes angiogenesis by secreting MMPs, plasminogen activators, and collagenase. Further, the interaction of FGFs with their high-affinity receptors activates intrinsic tyrosine kinase, which is involved in inducing early gene transcription and cell proliferation [61]. Upon ligand binding, FGF receptors undergo dimerization and transphosphorylation at tyrosine residues. Both bFGF and VEGF165 interact with heparin sulfate (HS) through tyrosine kinase receptors, resulting in the induction of a proliferative signal that stabilizes the FGF-FGFR interaction. The modulation of bFGF binding to high-affinity cell surface receptor sites can be achieved by heparin-mimicking compounds like RG-13577 [62]. These compounds disrupt abnormal bFGF signaling by competing with HS binding. Proteolytic enzymes, such as MMP-2, also play a role by cleaving the ectodomain of the receptor, thereby inhibiting bFGF-mediated signal transduction and EC proliferation [63].

FGF signaling serves as a critical regulator of both blood and lymphatic vascular development, influencing endothelial metabolism, which is essential for processes such as sprouting, proliferation, and migration [64]. The activation of the proangiogenic FGF signaling pathway is proposed as a mechanism employed by tumor cells to evade the effects of VEGF-targeted therapies. A recent study demonstrated that the inhibition of FGF receptors leads to a reduction in vessel density and restoration of tumor sensitivity to anti-VEGF therapy in a murine breast cancer model [57,65]. Findings from this study underscore the potential significance of targeting FGF signaling in overcoming resistance to antiangiogenic treatments in certain cancer scenarios.

#### 2.1.3. Platelet-Derived Growth Factor (PDGF)

The PDGF ligand family, 30 kDa dimer, consists of four structurally related soluble heparin-binding polypeptide growth factors labelled as PDGF- A, B, C, and D, forming five different homodimers and heterodimers viz. PDGF-AA, PDGF-BB, PDGF-AB, PDGF-CC, and PDGF-DD [66,67]. PDGF is secreted by activated platelets, endothelial cells, epithelial cells, glial cells, and inflammatory cells and targets mesoderm-derived cells such as pericytes, fibroblasts, glial cells, smooth muscle cells, or mesangial cells. PDGF signals through two Class III cell-surface tyrosine kinase receptors, PDGFRα and PDGFRβ, to regulate tumor angiogenesis. PDGF plays a key role in vessel maturation, pericyte recruitment, wound healing, and VEGF upregulation [68].

The members of the PDGF family display angiogenic activity in animal models, characterizing the PDGF-B/PDGFRβ axis in vascular development. PDGF-B is generated by developing and quiescent ECs, while PDGFRβ is expressed by perivascular cells and ECs. Lacking components of the PDGF-B/PDGFRβ pathway in mice leads to vessel leakage and microhemorrhages [69], highlighting the significance of PDGF in maintaining proper vessel integrity and function.

In the realm of cancer development and progression, PDGF and PDGFR contribute through autocrine and paracrine stimulation of tumor cells and stromal cells, respectively. This cascade leads to the induction of tumor-associated angiogenesis. A study was conducted on experimental glioma models, which demonstrated that PDGF-B is involved in the enhancement of angiogenesis by VEGF stimulation in tumor-associated ECs. Additionally, it also contributed to the recruitment of pericytes in the newly formed vessels [70]. In another study, when tumor cells produced PDGF-B or PDGF-D in the B16 mouse melanoma model, it was correlated with an elevated presence of pericytes within the tumor, resulting in an increased tumor growth rate [71]. Overall, PDGF actively participates in pericyte recruitment during tumor angiogenesis.

In addition, the role of PDGF/PDGFR is evidenced in vascular development using knockout models. Mice lacking PDGF-B and PDGFRβ succumb perinatally to vascular defects observed in various organs. However, the precise cause of mortality remains unclear. In transgenic mice, deletion of either PDGFRα or PDGFRβ did not result in vascular defects, but deletion of both receptors disrupted yolk sac blood vessel development [72]. PDGFR expression plays a crucial role in the yolk sac mesothelium and blood vessel development. These findings emphasize the indispensable contribution of PDGF signaling to vessel growth, ECM deposition, and vascular remodeling [73].

#### 2.1.4. Angiopoietin

Angiopoietins (Angs) are growth factors that play a pivotal role in development, maintenance, blood vessel remodeling, tumor growth, and angiogenesis. The human angiopoietin family includes ligands Ang1, Ang2, and Ang4. Angs signal through endothelial receptor tyrosine kinases viz. Tie1 and Tie2 [74,75]. Both angiopoietin-1 (Ang1) and angiopoietin-2 (Ang2) act as proangiogenic and antiangiogenic factors, signaling through the Tie2 receptor [76,77]. Ang 2 is a competitive inhibitor of Ang 1. An in vivo study by Lobov et al. (2002) found that Ang2 facilitates the increase in capillary diameter, EC proliferation and migration, basal lamina remodeling, and new blood vessel sprouting in the presence of endogenous VEGF-A. Conversely, the inhibition of endogenous VEGF activity leads to EC death and vessel regression by Ang 2 [78]. The paracrine activity of Ang1 induces the phosphorylation of Tie2 and the p85 subunit of PI 3′-kinase, leading to increased PI 3′-kinase activity in a dose-dependent manner and EC survival [79]. Additionally, Ang1 promotes vessel maturation and stabilization and prevents EC apoptosis through the Akt/survivin pathway. Furthermore, Ang1 induces EC sprouting by increasing plasmin and MMP-2 secretion and suppressing TIMP-2 secretion, whereas PI 3′-kinase inhibitors inhibit these processes as well as the Ang1-stimulated tyrosine phosphorylation of p125FAK [80]. Ang2 acts as a checkpoint, blocking Ang1-mediated Tie2 autophosphorylation in ECs, and induces vessel destabilization and pericyte detachment. This cascade of events prevents excessive branching and sprouting of blood vessels [81]. In glioblastoma, increased Ang2 expression has been associated with the reduced efficacy of anti-VEGF treatment and increased therapy resistance [82]. Preclinical studies have shown that the dual inhibition of Ang2/VEGFR2 yields beneficial effects on inhibiting tumor progression. Thus, the dynamic interplay between angiopoietins and their receptors contributes to the complex regulation of angiogenesis in both physiological and pathological contexts [83,84].

#### 2.1.5. Matrix Metalloproteases (MMPs)

MMPs play a pivotal role in inducing tumor angiogenesis by ECM degradation, and the release of angiogenic mitogens. Notably, MMP-9 and MMP-2 are key contributors, as they proteolytically cleave and activate latent transforming growth factor-beta (TGF-β), thereby promoting tumor angiogenesis [85]. The expression of MMPs aids in ECM remodeling during tumor progression, thereby releasing ECM- and membrane-bound growth factors involved in tumor progression, metastasis, and tumor-associated angiogenesis. The transcription of MMPs can be regulated by cytokines, growth factors, and mechanical stress [86]. Interleukin-8 (IL-8), a pro-inflammatory cytokine, induces MMP-2 production involved in the induction of angiogenesis. When the tumor cells are transfected with IL-8, it results in the upregulation of MMP-2 mRNA levels while VEGF and bFGF mRNA levels remain unchanged [87,88]. Beyond their role in ECM degradation, MMP-2 and MMP-3 exhibit additional capabilities of releasing soluble FGFR1 and soluble 12-kDa immunoreactive and mitogenic heparin-binding epidermal growth factor (HB-EGF), respectively. MMP-2 directly modulates melanoma cell adhesion, ECM spreading, and invasion, while its inhibitors inhibit growth and tumor neovascularization by preventing MMP-2 binding to αvβ3 and blocking cell surface collagenolytic activity [89]. The intricate interplay between MMPs, cytokines, and growth factors highlights their multifaceted roles in the tumor microenvironment.

#### 2.1.6. Interleukins

**Figure 2:**
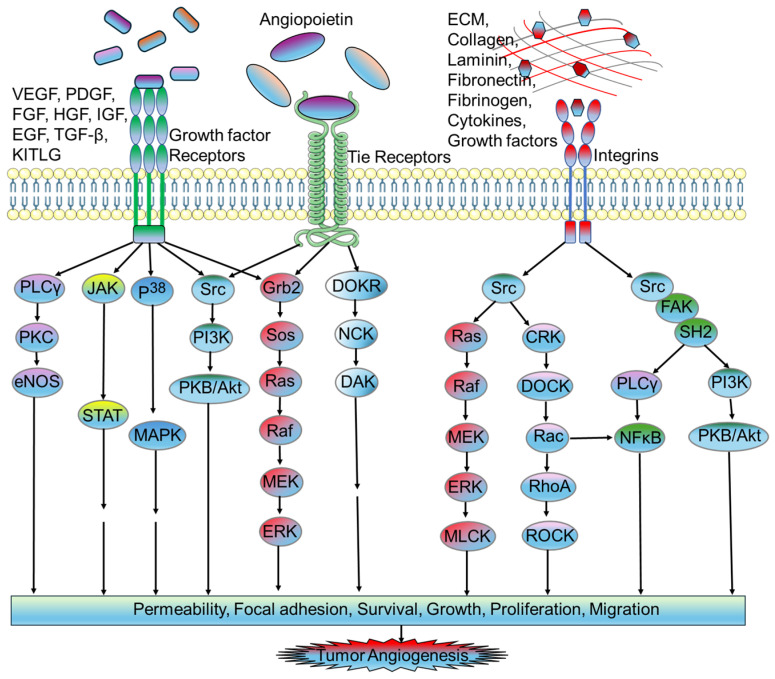
Proangiogenic signaling pathways in endothelial cells associated with tumor microenvironment. Activation of growth factor receptors with specific ligands (e.g., VEGF, PDGF, FGF, HGF, IGF, EGF, TGF-β and KITLG) triggers the activation of different downstream signaling pathways, including the PLCγ/PKC/eNOS, JAK/STAT, P^38^/MAPK, Src/PI3K/Akt, and Grb2/Sos/Ras/Raf/MEK/ERK signaling pathways. The activation of Tie receptors upon angiopoietin binding leads to the activation of intracellular signaling pathways, including the Src/PI3K/Akt, Grb2/Sos/Ras/Raf/MEK/ERK, and DOKR/NCK/DAK signaling pathways. The activation of integrins with specific ligands (e.g., ECM, collagen, laminin, fibronectin, fibrinogen, cytokines, and growth factors) leads to the activation of the downstream effector, Src, which in turn activates several signaling pathways, including the Ras/Raf/MEK/ERK/MLCK, CRK/DOCK/Rac/RhoA/ROCK, FAK/SH2/PLCγ/NFκB, or FAK/SH2/PI3K/Akt signaling pathways. All of these signaling pathways result in tumor angiogenesis by increasing cellular permeability, focal adhesion, survival, growth, proliferation, and migration. Abbreviations: VEGF, vascular endothelial growth factor, PDGF, platelet-derived growth factor; FGF, fibroblast growth factor; HGF, hepatocyte growth factor; IGF, insulin-like growth factor; EGF, epidermal growth factor; TGF-β, transforming growth factor beta; KITLG, KIT ligand; ECM, extracellular matrix; PLCγ, phospholipase C gamma; PKC, protein kinase C; eNOS, endothelial nitric oxide synthase; JAK, janus kinase; STAT, transducer and activator of transcription; MAPK, mitogen-activated protein kinases; Src, PI3K, phosphoinositide 3-kinases; PKB, protein kinase B; Grb2, growth factor receptor bound protein 2; Sos, son of sevenless; Ras, rat sarcoma virus; Raf, rapidly accelerated fibrosarcoma; MEK, mitogen-activated protein kinase kinase; ERK, extracellular signal-regulated kinase; DOKR, downstream of kinase-related protein; NCK, noncatalytic region of tyrosine kinase; DAK, dihydroxyacetone kinase; MLCK, myosin light-chain kinase; CRK, CT10-regulated kinase; DOCK, dedicator of cytokinesis; Rac, ras-related C3 botulinum toxin substrate; RhoA, ras homolog family member A; ROCK, rho-associated protein kinase; NFκB, nuclear factor kappa B; FAK, focal adhesion kinase; SH2, src homology 2.

Interleukins (ILs) are a group of cytokines that play crucial roles in regulating immune responses and inflammation. In the context of tumors, certain interleukins have been found to possess proangiogenic properties. Several interleukins have been implicated in promoting angiogenesis within the tumor microenvironment. IL-1 isoforms, particularly Interleukin-1β (IL-1β), have been implicated in promoting angiogenesis by stimulating the production of proangiogenic factors such as VEGF, IL-8, and basic fibroblast growth factor (bFGF). IL-1 can also induce the expression of adhesion molecules on endothelial cells, facilitating their interaction with circulating leukocytes and promoting angiogenesis [90]. IL-6 has been shown to promote angiogenesis indirectly by stimulating the production of VEGF and other angiogenic factors from both tumor cells and stromal cells within the tumor microenvironment. Additionally, IL-6 can enhance the survival and proliferation of endothelial cells, thereby facilitating the formation of new blood vessels [90,91]. IL-8 is a member of the chemokine family. It binds with chemokine receptors (CXCR1 and CXCR2) with high affinity. IL-8 is a potent angiogenic factor that stimulates endothelial cell migration and proliferation, contributing to the formation of new blood vessels within tumors [92,93]. IL-17 is a proinflammatory cytokine that has been shown to promote angiogenesis in various tumors. It can induce the expression of proangiogenic factors such as VEGF, IL-8, and matrix metalloproteinases (MMPs), which facilitate endothelial cell migration and vessel formation [94,95]. IL-23, primarily produced by activated dendritic cells and macrophages, has been implicated in promoting angiogenesis by inducing the production of proangiogenic factors such as VEGF and IL-6. IL-23 can also enhance the recruitment and activation of inflammatory cells within the tumor microenvironment, further promoting angiogenesis [95,96] (Figure 2).

### 2.2. Antiangiogenic Factors in Tumor Microvasculature

#### 2.2.1. Endostatin

**Figure 3:**
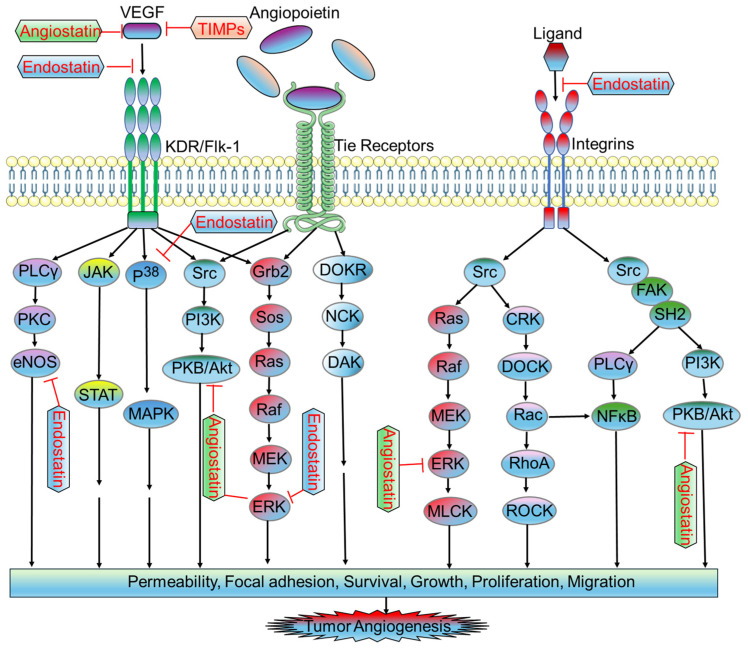
Antiangiogenic signaling pathways in endothelial cells associated with tumor microenvironment. The activation of growth factor receptors with specific ligands (e.g., VEGF) triggers the activation of different downstream signaling pathways, including the PLCγ/PKC/eNOS, JAK/STAT, P^38^/MAPK, Src/PI3K/Akt, and Grb2/Sos/Ras/Raf/MEK/ERK signaling pathways. The activation of Tie receptors upon angiopoietin binding leads to the activation of intracellular signaling pathways, including the Src/PI3K/Akt, Grb2/Sos/Ras/Raf/MEK/ERK, and DOKR/NCK/DAK signaling pathways. The activation of integrins with specific ligands leads to the activation of the downstream effector, Src, which in turn activates several signaling pathways, including the Ras/Raf/MEK/ERK/MLCK, CRK/DOCK/Rac/RhoA/ROCK, FAK/SH2/PLCγ/NFκB, or FAK/SH2/PI3K/Akt signaling pathways. All of these signaling pathways result in tumor angiogenesis by increasing cellular permeability, focal adhesion, survival, growth, proliferation, and migration. The endogenous antiangiogenic factor, endostatin, prevents the binding of VEGF with KDR/Flk-1 by directly occupying the binding site of VEGF on KDR/Flk-1. Other endogenous antiangiogenic factors, including angiostatin and TIMPs, also inhibit KDR/Flk-1 activation by downregulating the generation of VEGF. Endostatin inhibits integrin signaling by sequestering the binding of specific ligands with integrin. It also inhibits P38/MAPK and ERK signaling, as well as the activation of eNOS. Angiostatin also inhibits Akt and ERK activation. Abbreviations: VEGF, vascular endothelial growth factor; TIMPs, tissue inhibitors of metalloproteinases; KDR, kinase insert domain receptor; Flk-1, fetal liver kinase 1; PLCγ, phospholipase C gamma; PKC, protein kinase C; eNOS, endothelial nitric oxide synthase; JAK, Janus kinase; STAT, transducer and activator of transcription; MAPK, mitogen-activated protein kinases; Src, PI3K, phosphoinositide 3-kinases; PKB, protein kinase B; Grb2, growth factor receptor bound protein 2; Sos, son of sevenless; Ras, rat sarcoma virus; Raf, rapidly accelerated fibrosarcoma; MEK, mitogen-activated protein kinase kinase; ERK, extracellular signal-regulated kinase; DOKR, downstream of kinase-related protein; NCK, noncatalytic region of tyrosine kinase; DAK, dihydroxyacetone kinase; MLCK, myosin light-chain kinase; CRK, CT10-regulated kinase; DOCK, dedicator of cytokinesis; Rac, ras-related C3 botulinum toxin substrate; RhoA, ras homolog family member A; ROCK, rho-associated protein kinase; NFκB, nuclear factor kappa B; FAK, focal adhesion kinase; SH2, src homology 2.

Endostatin is a 20 KDa fragment derived from type XVIII collagen. It is a naturally occurring protein that acts as an inhibitor of EC proliferation, migration, angiogenesis, and tumor growth, exerting its antiangiogenic effects in the formation of new blood vessels [97]. It has a multifaceted mode of action that disrupts key pathways associated with VEGF signaling. Notably, endostatin inhibits the binding of VEGF to ECs, interacts with the KDR/Flk-1 receptor, and blocks the VEGF-induced tyrosine phosphorylation and KDR/Flk-1 events [98]. Additionally, it suppresses downstream signaling events involved in VEGF mitogenic and motogenic effects in ECs, including the activation of ERK, p38 MAPK, and p125FAK. Endostatin also exhibits anti-migratory effects by reducing VEGF-induced eNOS phosphorylation [99] (Figure 3).

Furthermore, endostatin inhibits integrin functions such as EC migration, by binding to αv- and α5-integrins. It competes with bFGF for binding to HSPGs on the cell surface, disrupting mitogenic growth factor signaling [100]. Additionally, endostatin exerts anti-proliferative effects by inhibiting MMP-2 activation and activity, hindering the invasiveness of ECs and tumor cells [101]. Importantly, the proapoptotic activity of endostatin reduces antiapoptotic proteins (bcl-2 and bcl-XL) without affecting the proapoptotic Bax protein. The Shb adaptor protein is implicated in mediating the apoptotic signaling of endostatin [102]. The ability of endostatin to target multiple facets of VEGF signaling and cellular processes positions it as a valuable candidate in the pursuit of effective anti-cancer strategies.

#### 2.2.2. Angiostatin

Angiostatin is a 38 KDa internal fragment derived from plasminogen. It exhibits potent antiangiogenic effects primarily attributed to its ability to downregulate VEGF expression within tumors [103]. This internal fragment interacts with the plasma membrane-localized ATP synthase, thereby suppressing endothelial-surface ATP metabolism. This process leads to the downregulation of EC proliferation and migration, contributing to the overall antiangiogenic effects of angiostatin [104]. Studies have shown that angiostatin treatment not only significantly increases apoptosis of ECs but also inhibits hepatocyte growth factor (HGF)-induced phosphorylation of c-met, Akt, and ERK1/2 (Figure 3). This disruption of HGF/c-met signaling is a crucial mechanism through which angiostatin exerts its antiangiogenic effects [105,106]. The intraperitoneal administration of angiostatin has been demonstrated to robustly inhibit neovascularization and metastasis in mice following the removal of a primary tumor [107].

The downregulation and inhibition of EC proliferation and migration are also attributed to the binding of angiostatin to the α/β-subunits of plasma membrane-localized ATP synthase and adenoviral-mediated angiostatin gene transfer by disrupting the G2/M transition, thereby resulting in the suppression of endothelial-surface ATP metabolism and M-phase phosphoproteins, respectively [108,109]. Angiostatin induces a transient increase in ceramide levels, correlating with actin stress fiber reorganization, cell detachment, and death. Additionally, angiostatin or ceramide treatment activates RhoA and E-selectin to inhibit EC proliferation, further contributing to its antiangiogenic effects. The multifaceted actions of angiostatin, from downregulating VEGF expression to disrupting cell signaling pathways and inducing apoptosis, highlight its potential as a promising antiangiogenic agent in tumor growth and metastasis [110].

#### 2.2.3. Tissue Inhibitors of Metalloproteinases (TIMPs)

TIMPs are involved in the regulation of the dynamic process of ECM remodeling, influencing EC behavior, and impacting neovascularization. The remodeled ECM provides a scaffold for EC adhesion, migration, and tube formation, while these components are deposited to form the basal lamina surrounding endothelial and mural cells. The overexpression of TIMP-1 has been shown to significantly inhibit in vitro migration of ECs through gelatin, while TIMP-2 has been reported to inhibit bFGF-induced EC proliferation [111]. Furthermore, TIMP-2 exhibits the ability to inhibit the release of soluble FGFR1 by MMP-2, a mechanism that contributes to its antiangiogenic effects. The transfection of highly metastatic melanoma cells with TIMP-2 cDNA resulted in reduced blood vessel formation, and diminished EC migration and invasion [112]. TIMP-3, another member of the TIMP family, has been associated with inducing apoptotic cell death in various cancer cell lines and rat vascular smooth muscle cells. This effect is attributed to the stabilization of TNF-alpha receptors on the cell surface by inhibiting MMPs. TIMP-3 also exhibits antiangiogenic and antitumor effects by downregulating vascular endothelial cadherin expression, highlighting the diverse impacts of TIMP-3 on neovascularization and tumor progression [113,114].

Importantly, TIMP-1, TIMP-2, TIMP-3, and TIMP-4 collectively contribute to the inhibition of neovascularization by impeding the breakdown of the surrounding matrix induced by MMPs such as MMP-1, MMP-2, and MMP-9. TIMPs have the ability to influence both endothelial and tumor cell migration. The degradation of the basement membrane and remodeling of the ECM, regulated by the interplay between MMPs and TIMPs, is essential for creating a conducive environment for endothelial cell migration and proliferation [115,116].

#### 2.2.4. Interferons and Interleukins

Interferons (IFNs), including IFN-α, IFN-β, and IFN-γ, are secreted glycoproteins that exhibit inhibitory effects on tumor angiogenesis. IFN-α/β downregulates the MMP-9 expression at both mRNA and protein levels [117]. Additionally, they inhibit IL-8 expression, demonstrating their antiangiogenic properties in bladder cancer. IFN-α/β administration has also been associated with a decrease in mRNA expression and protein level of bFGF, thereby reducing microvessel density in tumors and inducing EC apoptosis. However, the regulation of angiogenic factors by IFNs appears to be tumor-specific due to its varying effects on bFGF and VEGF levels in renal cell carcinomas, carcinoid tumors, and leukemia patients [118]. IFN-γ induces antiangiogenic effects through the secretion of inducible protein 10 (IP-10) and monokine. Beyond their antiangiogenic effects, IFNs also possess antitumor properties by upregulating major histocompatibility classes I and II, immunogenicity, and the activation of macrophages, T lymphocytes, and natural killer cells [119]. On the other hand, the angiogenic or antiangiogenic role of ILs is influenced by their specific structural features. For instance, IL-8 contains a Glu-Leu-Arg (ELR) motif at the NH2 terminus known to enhance angiogenesis, while IL-4 lacks this motif and inhibits angiogenesis, in vivo bFGF-induced neovascularization, and the in vitro migration of microvascular ECs. Conversely, IL-1α, secreted by activated macrophages, induces angiogenesis by upregulating VEGF, IL-8, and bFGF expressions. IL-6 counteracts the apoptotic effects mediated by wild-type p53 [120]. Further, IL-12 demonstrated antiangiogenic effects by suppressing the mRNA expression of VEGF, bFGF, and MMP-9. It stimulates the expression of IFN-γ and its inducible antiangiogenic chemokine IP-10 in ECs, promoting apoptosis and inhibiting proliferation in human tumors. This cascade of events leads to reduced tumor vessel density [121]. IL-10 has been associated with inhibiting neovascularization and tumor metastasis by downregulating the synthesis of VEGF, IL-1β, TNF-α, IL-6, and MMP-9 in tumor-associated macrophages and natural killer (NK) cell-dependent mechanisms, respectively [90].

## 3. Antiangiogenic Therapies

About 48 years ago, Judah Folkman and colleagues introduced the concept of targeting angiogenesis to impede tumor growth. Since then, antiangiogenic therapies focusing primarily on the regulatory factors and signaling pathways involved in tumor angiogenesis have been developed and approved for treating various types of tumors. Despite encouraging outcomes observed in preclinical studies, anti-VEGF monotherapies like bevacizumab, sunitinib, and aflibercept have demonstrated benefits in specific tumor types such as advanced-stage renal cell carcinoma, hepatocellular carcinoma, and colorectal carcinoma. However, these therapies have not proven effective in treating pancreatic adenocarcinoma, prostate cancer, breast cancer, or melanoma [122]. The goal for more effective antiangiogenic strategies continues to enhance treatment outcomes and expand the applicability of angiogenesis-targeted therapies across a broader spectrum of cancer types. Table 1 summarizes the possible antiangiogenic therapies with their potential targets, inhibitors, drugs, and mechanisms of action.

The role of combination therapies to enhance the effectiveness of antiangiogenic treatments is indeed crucial. For instance, the combination of anti-VEGF therapies with either chemotherapy or immunotherapy presents a promising approach to improve treatment outcomes by targeting multiple pathways involved in tumor growth and progression. However, despite the potential benefits of such combination strategies, resistance to antiangiogenic therapies remains a significant challenge in cancer treatment.

Several probable mechanisms and modes of resistance contribute to the development of resistance to antiangiogenic therapies. Tumors can activate compensatory angiogenesis pathways, such as the upregulation of alternative proangiogenic factors like fibroblast growth factor (FGF) or platelet-derived growth factor (PDGF), to bypass the inhibition of VEGF signaling [123]. Additionally, tumor hypoxia induced by antiangiogenic therapies can trigger adaptive responses leading to the recruitment of proangiogenic factors and the formation of new blood vessels via alternative mechanisms. Moreover, increased invasiveness and metastasis may occur as a result of antiangiogenic treatments, with hypoxia-driven changes in tumor cells enhancing their ability to invade surrounding tissues and disseminate to distant sites [124]. Furthermore, tumor heterogeneity plays a role in resistance, as some tumor cells may acquire resistance through genetic mutations or epigenetic changes that promote survival and growth despite antiangiogenic treatment. Additionally, tumor-associated stromal cells, such as cancer-associated fibroblasts and pericytes, can contribute to resistance by promoting angiogenesis and providing a supportive microenvironment for tumor growth (Azam 2010). Moreover, antiangiogenic therapies may modulate the tumor immune microenvironment, leading to immune evasion and resistance to immunotherapy. For example, VEGF inhibition can impair dendritic cell maturation and T cell function, limiting the effectiveness of immune checkpoint inhibitors [125]. Understanding these mechanisms of resistance is crucial for developing strategies to overcome resistance and improve the long-term efficacy of antiangiogenic therapies in cancer treatment. Combination therapies targeting multiple pathways involved in tumor angiogenesis and progression, as well as strategies to overcome immune evasion, hold promise in addressing resistance and improving patient outcomes.

## 4. Experimental Approaches to Determine Intricacies in Tumor Angiogenesis

To explore the molecular, cellular, and structural aspects of angiogenesis within the tumor microenvironment, several techniques are required to interpret the multidisciplinary aspects of tumor angiogenesis. The key techniques to understand the intricacies of tumor angiogenesis are discussed below.

### 4.1. Radionuclide-Based Imaging Modalities

In the past decade, radionuclide-based imaging became a routine practice in clinical settings due to the accessibility of gamma cameras (g-cameras). Single photon emission computed tomography (SPECT) and positron emission tomography (PET) scanners are more prevalent for the imaging of VEGFRs [126]. Various radioisotopes have been employed for imaging such as ^123^I, ^111^In, and ^99m^Tc for SPECT imaging, and ^64^Cu and ^89^Zr for PET applications [127]. This diverse array of radioisotopes allows for flexibility in choosing the most suitable imaging modality based on factors such as resolution, sensitivity, and specificity. For instance, the binding properties of 123I-VEGF165 and 123I-VEGF121 to human umbilical vein endothelial cells and different human tumor cell lines expressed a substantial number of VEGFRs in primary tumors and metastasis [128]. In two different studies, 123I-VEGF165 was used in patients with gastrointestinal cancer and pancreatic carcinomas and showed high receptor-binding affinity; however, it had low metabolic stability, and lesions were observed in a few patients [128,129]. 125I-VEGF121 and 125I-VEGF165 exhibit different accumulation patterns with higher 125I-VEGF165 uptake in several organs such as the heart, lungs, and kidneys. Both the monoclonal antibodies, i.e., VEGF-bound VG76e and HuMV833, were labeled with ^124^I for PET imaging of solid tumor xenografts in immunodeficient mice [130,131]. Bevacizumab, another monoclonal antibody, was labeled with both ^111^In for SPECT and ^89^Zr for PET imaging [132]. This dual-labeling approach allows for the utilization of both SPECT and PET modalities, providing a comprehensive assessment of the antibody’s biodistribution and targeting efficiency. The study involved nude mice xenografted with SKOV-3 human ovarian tumors. Subsequently, these mice were injected with either ^89^Zr-labeled bevacizumab, ^111^In-labeled bevacizumab, or ^89^Zr-labeled immunoglobulin G and showed tumor localization [133]. The use of both SPECT and PET imaging modalities provides complementary information, enhancing the overall understanding of the antibody’s performance in the tumor microenvironment. While these radionuclide-based imaging techniques are valuable in clinical practice, they have specific limitations that should be considered. For instance, SPECT imaging has a relatively lower spatial resolution compared to PET, which may limit its ability to detect small lesions or accurately localize VEGFR expression within the tumor. Additionally, the availability of specific radioisotopes for SPECT imaging may be limited, leading to challenges in clinical implementation [130]. PET imaging, although more sensitive and quantitative, is associated with higher costs and radiation exposure compared to SPECT. Furthermore, the short half-life of some PET radioisotopes necessitates on-site cyclotron facilities for their production, which may not be available at all medical centers. Looking ahead, advancements in radiotracer design and imaging technology may improve the sensitivity, specificity, and clinical utility of both SPECT and PET imaging modalities. Moreover, the development of novel VEGFR-targeted tracers and the integration of hybrid imaging systems, such as PET/CT and PET/MRI, may further enhance the accuracy and precision of VEGFR imaging in cancer diagnosis and management [134,135].

### 4.2. Non-Radionuclide-Based Imaging Modalities

Dynamic contrast-enhanced magnetic resonance imaging (DCE-MRI) is a noninvasive imaging technique employed to assess parameters associated with tissue perfusion and permeability, particularly in the context of examining tumor vasculature [136]. This imaging modality relies on the administration of contrast agents to visualize and study the dynamic changes in signal intensity over time. Another technique known as ultrasonography is the most commonly utilized clinical imaging modality. This technique is safe, cost-effective, user-friendly, and widely available [137]. The contrast in ultrasound images is influenced by factors such as sound speed, sound attenuation, backscatter, and the imaging algorithm employed. The microcirculation is visualized using Doppler and microbubble methods that quantify the relative fractional vascular volume and blood flow, respectively [138]. In tumor development or antiangiogenic cancer therapy, noninvasive ultrasound imaging results were compared with DCE-MRI results, correlating the imaging findings with the underlying histopathological changes [139]. In a study, contrast-enhanced ultrasound (CEU) imaging was used to image microbubbles after treatment with anti-VEGF monoclonal antibodies or a chemotherapy drug (gemcitabine) and showed significant enhancement of tumor vasculature [140]. Lastly, fluorescence imaging using Cy5.5 fluorescent dye to conjugate VEGF demonstrated tumor contrast; however, it is associated with a significant background signal, lack of whole-body distribution reports, and it is less quantitative [141]. In clinical practice, DCE-MRI and ultrasound imaging are often used together to complement each other’s strengths. For example, DCE-MRI can provide detailed anatomical information about the tumor vasculature, while ultrasound imaging can provide real-time monitoring during procedures such as biopsies or tumor ablations [142]. Research studies have also demonstrated the effectiveness of these imaging techniques in monitoring tumor angiogenesis and assessing treatment response [143,144]. Overall, non-radionuclide imaging techniques such as DCE-MRI and ultrasound imaging play a crucial role in monitoring tumor angiogenesis and assessing treatment response in cancer patients. Their ability to provide detailed information about tumor microvasculature makes them valuable tools in oncology research and clinical practice [145].

### 4.3. Biomarkers Assessment

Biomarkers, such as vascular endothelial growth factor (VEGF) and genetic variations in its receptors, are increasingly important in predicting clinical stages and prognoses for patients with different types of cancer. In clinical practice, these biomarkers play a crucial role in guiding personalized therapy selection, leading to improved patient outcomes. For instance, elevated levels of VEGF in serum or tissue samples are associated with more aggressive tumor behavior and poorer prognosis in various cancers, including colorectal, lung, breast, and renal cell carcinomas [146]. This biomarker is used to stratify patients into different risk groups and guide treatment decisions. In addition, genetic variations in VEGF receptors, such as VEGFR-2, can influence the response to anti-VEGF therapies. Patients with specific VEGFR-2 mutations may benefit more from anti-VEGF targeted therapies, such as bevacizumab, compared to those without these mutations [146,147]. In personalized medicine, biomarkers like VEGF and its receptors are used to tailor treatment strategies to individual patients, maximizing efficacy and minimizing side effects. For instance, patients with high VEGF levels may be more likely to benefit from anti-VEGF therapies, while those with specific VEGFR mutations may require different treatment approaches. By incorporating biomarker analysis into clinical practice, healthcare providers can optimize treatment selection and improve patient outcomes in cancer care [148]. Evidence in the literature shows that the expression of VEGF and its receptors are correlated with clinical stages and prognosis in tumor tissues [149]. In the immunohistochemical (IHC) analysis of breast cancers, an increased abundance of phosphorylated VEGFR2 (pVEGFR2) was observed. Following therapy with bevacizumab, there was a decrease in the amount of pVEGFR2, which is a finding that was also correlated with clinical efficacy [150]. This observation suggests that monitoring the phosphorylation status of VEGFR2 through IHC analysis can serve as a potential indicator of treatment response in breast cancer. In hepatocellular carcinomas (HCCs), the efficacy of sorafenib treatment was found to be more pronounced in patients with highly active mitogen-activated protein kinase (MAPK), as determined from the IHC staining of phosphorylated extracellular signal-regulated kinase (pERK). This study aims to provide further insights into the correlation between MAPK activity and the therapeutic response to sorafenib in hepatocellular carcinoma [151].

Bv8, also known as prokineticin 1 (PROK1), is a protein related to the endocrine gland-derived VEGF. Functional analyses of Bv8 have revealed its ability to induce the mobilization of myeloid cells from the bone marrow and, notably, to circumvent VEGF-mediated angiogenesis. This suggests that Bv8 plays a role in modulating the recruitment and movement of myeloid cells, potentially influencing the dynamics of the immune response and angiogenic processes in the body. The identification and characterization of Bv8 highlight its significance in the complex regulatory network governing vascular and immune functions [152,153].

In metastatic colorectal cancer, Kopetz et al. conducted a study analyzing a panel of numerous circulating angiogenic factors (CAFs), encompassing up to 40 angiogenic and immunological markers at defined time points [154]. The investigation was carried out during FOLFIRI plus bevacizumab therapy. The results demonstrated that several measured cytokines, such as bFGF, PlGF, MMP-9, and HGF or PDGF, exhibited alterations during the course of therapy. The finding from this study suggests that the levels of circulating angiogenic factors in the bloodstream could serve as predictive indicators of resistance to antiangiogenic therapies [155].

**Figure 4:**
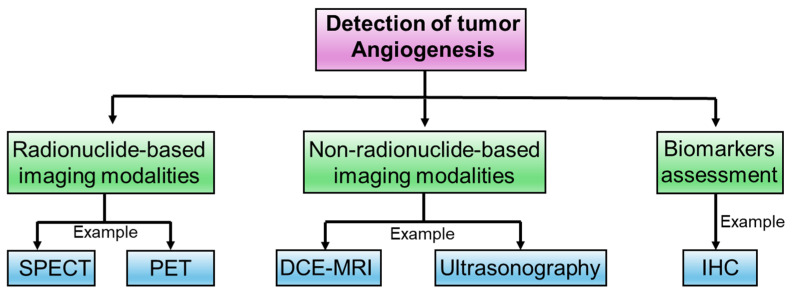
Different strategies to detect tumor angiogenesis. Abbreviations: SPECT, single photon emission computed tomography (SPECT); PET, positron emission tomography; DCE-MRI, dynamic contrast-enhanced magnetic resonance imaging; IHC, immunohistochemistry.

Elevated levels of circulating endothelial cells (CECs) and circulating progenitor cells (CEP) have been linked to various diseases, including myocardial infarction, transplant reactions, and cancer [156]. The presence of elevated CEC levels in the bloodstream of individuals with cancer implies a potential role for these cells as biomarkers that could be indicative of vascular changes associated with malignancies. Monitoring CEC and CEP counts may contribute to the diagnosis and assessment of vascular involvement in cancer patients [157,158] (Figure 4).

## 5. Conclusions

The study of tumor angiogenesis provides insights into the understanding of cancer biology and therapeutic strategies. The regulation between proangiogenic and antiangiogenic factors within the tumor microenvironment highlights the complexity and multifaceted nature of tumor angiogenesis. From the initial recognition of angiogenesis as a hallmark of tumor progression to the development of targeted antiangiogenic therapies, a shift in the technological advancements in the intricacies of the tumor microenvironment has been evidenced. Emerging technologies such as single-cell analysis, advanced imaging modalities, artificial intelligence, and innovative therapeutic approaches pave the way for more effective, targeted, and personalized approaches to combat tumor angiogenesis.

## Figures and Tables

**Table 1:** Antiangiogenic therapies.

Targets	Inhibition Sites	Drugs	Mechanisms
VEGF signaling	Anti VEGF mAb	Bevacizumab, Aflibercept	Directly neutralizes the VEGF proteins
	Inhibitors of VEGF receptors	Sunitinib, Sorafenib	Bind to VEGF receptors
	Inhibitors of VEGF signal transduction pathway	LY294002, Wortmannin, FARA-A, Rapamycin, Temsirolimus, Everolimus, Tipifarnib, Lonafarnib.	Blocking autophosphorylation of VEGF receptors
	VEGF antisense		- bind to VEGF mRNA - interferes with the translation process - blocks the VEGF protein formation
EGFR signaling	Inhibitor of EGFR	Cetuximab, Panitumumab, Necitumumab	Block EGFR formation
RTK signaling	Tyrosine kinase inhibitor	Sunitinib, Sorafenib	Activity against VEGFR, PDGFR, Flt-3, C-kit & RET, CSF 1R
PI3K/Akt/mTOR signaling pathway	Inhibitors of PI3K pathway	LY294002 and wortmannin	
	Inhibitors of mTOR	Temsirolimus (CCI-779) and Everolimus (RAD001)	
MAPK signalling	Antiangiogenic therapy	Tipifarnib (R115777), Lonafarnib (SCH66336)	MAPK-Farnesyltransferase Rho and Ras
Other drugs	Methionine aminopeptidase inhibitor	Tnp-470	Prevents endothelial activation and arrest cell cycle
		Thalidomide	Inhibits TNF-α synthesis

Abbreviations: VEGF, vascular endothelial growth factor; mAb, monoclonal antibody; EGFR, epidermal growth factor receptor; RTK, receptor tyrosine kinase; PDGFR, platelet-derived growth factor; Flt-3, fetal liver kinase 3; CSF1R, colony stimulating factor 1 receptor; MAPK, mitogen-activated protein kinase; PI3K, phosphoinositide 3-kinases; Akt, protein kinase B; mTOR, mammalian target of rapamycin; TNF-α, tumor necrosis factor α; Rho, ras homolog family member; Ras rat sarcoma virus.

## Data Availability

Data available in a publicly accessible repository.
